# Ginsenoside Rb1 From the Functional Food *Panax ginseng* Suppresses Pancreatic Cancer Progression via Macrophage Reprogramming Through the PI3K‐AKT Pathway

**DOI:** 10.1002/fsn3.72468

**Published:** 2026-10-08

**Authors:** Chang‐Sheng Dong, Gaoqiang Wang, Ying Niu, Xiuqiao Sun, Wenyi Ren, Jiliang Zhang, Yi Zhang, Rui‐Xin Wu, Xiaoxiong Wu

**Affiliations:** ^1^ Cancer Institute, Department of Oncology Longhua Hospital, Shanghai University of Traditional Chinese Medicine Shanghai China; ^2^ Faculty of Chinese Medicine Macau University of Science and Technology Foundation Taipa Macau China; ^3^ Department of Traditional Chinese Medicine Internal Medicine Shanghai Baoshan Luodian Hospital Shanghai China; ^4^ Shanghai Tenth People's Hospital, Tongji University School of Medicine Shanghai China; ^5^ Preclinical Department Shanghai Municipal Hospital of Traditional Chinese Medicine, Shanghai University of Traditional Chinese Medicine Shanghai China; ^6^ Department of Traditional Chinese Medicine, Yangpu Hospital, School of Medicine Tongji University Shanghai China

**Keywords:** cancer research, functional food, ginseng, macrophage polarization, nutritional interventions, pancreatic cancer

## Abstract

Pancreatic ductal adenocarcinoma (PDAC) is a highly malignant gastrointestinal tumor, and its nutritional intervention strategies remain relatively limited. Ginseng, a representative medicinal herb officially included in China's “Food and Medicinal Same Origin” catalogue, is widely used in tea beverages, soups, and functional foods. Its active component, ginsenoside Rb1 (GRB1), derived from the medicine‐food homology plant *Panax ginseng* , has been reported to possess antitumor and immunomodulatory effects, but its role in the PDAC tumor microenvironment remains unclear. This study aims to screen and identify GRB1 from the Chai‐Gui‐Xiaoji formula containing ginseng, experimentally validate its antitumor mechanism against PDAC, and reveal its potential as a functional food ingredient or nutritional adjuvant derived from ginseng. This work is framed within the medicine–food homology (yao shi tong yuan) paradigm, an ancient philosophy holding that many edible plants possess both nutritional value and bioactive properties, which is increasingly recognized by modern nutritional science as a framework for developing evidence‐based functional food ingredients. sUsing ultra‐high performance liquid chromatography‐quadrupole time‐of‐flight mass spectrometry (UPLC‐Q‐TOF‐MS) technology, we screened bioactive components from Chaigui Xiaoji Formula (CGXJF) containing ginseng. Combining component analysis, blood absorption component analysis, and network pharmacology target screening, we identified ginsenoside Rb1 (GRB1) as the core candidate. Through target prediction, protein interaction analysis, KEGG enrichment, and molecular docking, we identified the PI3K‐AKT signaling pathway as a potential GRB1‐related pathway. Pancreatic ductal adenocarcinoma (PDAC) was established through in situ and liver metastasis mouse models, and functional validation was performed using macrophage and patient‐derived organoid models. Tumor growth and liver metastasis were evaluated through in situ and liver metastasis models; PI3K/AKT phosphorylation and M2‐like macrophage polarization were detected by immunoblotting and other methods. To explore the mechanistic basis of GRB1‐related tumor inhibition, we used macrophage function experiments and patient‐derived organoid models as complementary functional tools to evaluate the involvement of macrophage regulation. Following the screening logic of the medicine–food homology paradigm, priority was given to orally relevant bioactive constituents derived from edible botanicals with established dietary exposure. Network pharmacology identified 110 shared targets between GRB1 and pancreatic cancer from 587 candidate GRB1 targets and 905 PDAC‐related targets; the PPI network contained 491 nodes and 3728 edges, with hub targets including STAT3, AKT1, CASP3, BCL2, HIF1A, MTOR, and NFκB1, and KEGG enrichment locked PI3K‐AKT signaling as the priority pathway. In orthotopic and liver metastasis mouse models, GRB1 inhibited tumor growth, reduced Ki67‐positive proliferation, decreased liver metastatic burden, lowered Zeb‐1 and Vimentin expression, and prolonged survival without obvious toxicity. GRB1 treatment was associated with dose‐dependent reductions in PI3K and AKT phosphorylation and reduced M2‐like macrophage polarization, as shown by decreased CD206, CD163, ARG1, CD115, and TGFB1; molecular docking suggested possible interactions between GRB1 and AKT1 and PIK3CA. In macrophage models, GRB1 reduced M2‐like polarization and PI3K/AKT phosphorylation; conditioned medium from GRB1‐treated M2‐like macrophages reduced patient‐derived organoid growth, and co‐injection with GRB1‐treated M2‐like macrophages reduced metastatic burden in vivo. These findings suggest that GRB1 may modulate the immunosuppressive PDAC microenvironment partly through PI3K‐AKT‐associated macrophage modulation. An integrated UPLC‐Q‐TOF‐MS and network pharmacology strategy identified GRB1 from the medicine‐food homology herb *Panax ginseng* as the principal anti‐PDAC constituent. GRB1 exhibited anti‐PDAC efficacy, accompanied by attenuated PI3K‐AKT signaling and reduced M2‐like macrophage polarization, supporting its development as a ginseng‐derived functional food ingredient or nutritional adjuvant for PDAC prevention and supportive care. These findings exemplify how the medicine–food homology paradigm can bridge traditional dietary wisdom with modern nutritional oncology.

## Graphical abstract

Food‐derived bioactive compound ginsenoside Rb1 (GRB1) inhibits pancreatic cancer progression by reprogramming tumor‐associated macrophages. GRB1 suppresses the PI3K–AKT signaling pathway, thereby blocking M0‐to‐M2 macrophage polarization and reducing the accumulation of immunosuppressive macrophages within the tumor microenvironment. Graphical Abstract was created with MedPeer (medpeer.cn).

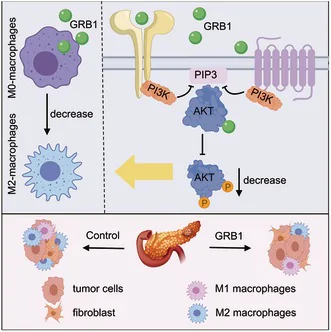

## 1 Introduction

Pancreatic ductal adenocarcinoma (PDAC) is one of the most aggressive human malignancies and has a 5 year survival rate of less than 10% (Blackford et al. 2024; Siegel et al. 2026). Because early detection remains difficult and disease progression is rapid, many patients present with locally advanced or metastatic disease at diagnosis (Halbrook et al. 2023; Nené et al. 2023). Even after surgical resection, recurrence is common and long‐term prognosis remains poor (van Goor et al. 2024). Epidemiological evidence indicates that dietary patterns, nutritional status, and metabolic disorders such as obesity and Type 2 diabetes are closely associated with PDAC risk and prognosis (Lu et al. 2025; Pan et al. 2020), making diet‐derived bioactive compounds an attractive strategy for its prevention and adjuvant management. This interest is deeply rooted in the concept of medicine–food homology, which holds that food‐derived bioactive compounds can modulate physiological functions through multi‐target, moderate‐affinity interactions compatible with chronic dietary intake. While the pharmacological effects of GRB1 are well documented, its potential as a daily dietary bioactive compound—consumed at lower, food relevant concentrations—remains underexplored.

*Panax ginseng* C. A. Meyer (ginseng) is one of the most widely consumed medicinal and edible plants in East Asia (Chen et al. 2021). In China, ginseng has been officially included in the catalogue of “substances that are traditionally used as both food and Chinese medicinal materials” (the medicine–food homology catalogue), permitting its use as a common food ingredient. As a food, ginseng is traditionally stewed in soups, brewed as tea, or processed into beverages, candies, and functional food products, and is valued for relieving fatigue, supporting immune function, and improving metabolic homeostasis; as a medicine, it has been used for centuries to “Yuan qi” and restore vital energy, and modern studies attribute these effects mainly to ginsenosides, ginseng polysaccharides, and other bioactive constituents with immunomodulatory, anti‐inflammatory, metabolic‐regulatory, and neuroprotective activities (Kim et al. 2018; Li et al. 2021; Ni et al. 2022; P. Zhou et al. 2019). This dual food–medicine identity makes ginseng and its constituents promising candidates for nutrition‐based cancer prevention and supportive therapy. Such officially recognized dual use as both food and medicine positions ginseng as a candidate functional food rather than merely a pharmaceutical agent, embodying the practical value of the medicine–food homology catalogue. Research has shown that the content of ginsenoside Rb1 varies under different farm conditions, ranging from 2.22 mg/g to 11.22 mg/g (Chen et al. 2019a).

To identify the anti‐PDAC active substance basis of ginseng‐containing preparations, we first applied ultra‐performance liquid chromatography–quadrupole time‐of‐flight mass spectrometry (UPLC‐Q‐TOF‐MS) to profile the constituents of the ginseng‐containing Chai‐Gui‐Xiao‐Ji Formula (CGXJF), an anti‐pancreatic‐cancer herbal formula developed by our group, and ginsenoside Rb1 (GRB1) was identified as one of its core bioactive components (Sun et al. 2025). We further compared GRB1 with other representative ginsenosides (e.g., Rg1, Re, and Rg3) in terms of relative abundance and reported antitumor evidence, and GRB1 was prioritized because of its high content in ginseng, favorable safety profile as a food‐derived saponin, and documented antitumor and immunomodulatory activities (Feng et al. 2024; Gao et al. 2020; Ni et al. 2022; P. Zhou et al. 2019). On this basis, a network pharmacology screen integrating three target‐prediction platforms (Swiss Target Prediction, SuperPred, and PharmMapper) with five PDAC disease‐target databases was performed to narrow down the candidate targets and pathways of GRB1, which finally locked the PI3K‐AKT signaling pathway as the priority candidate for experimental validation. This component‐to‐pathway screening strategy is consistent with the research logic of the medicine–food homology paradigm, which prioritizes bioactive constituents with a long history of safe dietary exposure.

Network pharmacology provides a hypothesis‐generating framework for exploring the multi‐target actions of natural products and food‐derived bioactive compounds in cancer therapy (Gao et al. 2020; Wu et al. 2024; Z. Zhang et al. 2022). As described above, GRB1 was screened out and locked as the core ginseng‐derived candidate. However, whether GRB1 modulates the immunosuppressive tumor microenvironment (TME) in PDAC remains unclear (Gou et al. 2023; Liu et al. 2023; Ni et al. 2022; Wang et al. 2023). Framed within the medicine–food homology paradigm, this question concerns not only pharmacological efficacy but also the nutritional relevance of a food‐derived compound.

Previous studies have shown that GRB1 can suppress colorectal cancer liver metastasis by inhibiting pro‐tumorigenic macrophages characterized by high SPP1 and CD206 expression (J. Zhou et al. 2024). These findings suggest that GRB1 may also affect pancreatic cancer progression through macrophage‐related mechanisms. However, direct evidence in PDAC remains limited.

In this study, we combined target prediction, protein–protein interaction analysis, KEGG enrichment, molecular docking, and experimental validation in mouse, macrophage, and organoid‐related models. We aimed to determine whether GRB1 suppresses PDAC progression and whether its effects are associated with PI3K‐AKT signaling and macrophage polarization within the TME, thereby evaluating the potential value and mechanism of this medicine–food homology‐derived compound as a functional food ingredient and nutritional adjuvant against PDAC. The study was therefore designed within the conceptual framework of medicine–food homology and functional food science.

## 2 Materials and Methods

### 2.1 Cell Culture, Antibodies and Reagents

Human cell lines (THP‐1, PANC‐1, PATU‐8988, ASPC‐1, CFPAC, MiaPaCa‐2, and HPNE) were obtained from the American Type Culture Collection (ATCC) and cultured in Dulbecco's Modified Eagle's medium (Meilunbio, China) with 10% FBS (Meilunbio, China) and 1% penicillin–streptomycin at 37°C under 5% CO_2_.

Gemcitabine (Solarbio, China, G8970), Ginsenoside Rb1 (Macklin, Shanghai, G742231), Mouse Ki67 (Abclonal, China, A20018); CK19 (Abclonal, China, A25546); SOX2 (Abclonal, China, A19118); Vimentin (Abclonal, China, A19607); Zeb‐1 (Abclonal, China, A5600); E‐cadherin (Abcam, USA, ab40772); AKT (Abclonal, China, AP0637); P‐AKT (Abclonal, China, AP0637); PI3K (Abclonal, China, A27717); P‐PI3K (Abclonal, China, A27717); GAPDH (Abclonal, China, A19056); CD45 (BioLegend, USA, 103106); CD3 (BioLegend, USA, 100219); CD11b (BioLegend, USA, 101224); CD206 (Abclonal, China, A21014); CD206 (BioLegend, USA, 141721); CD163 (BioLegend, USA, 155327); ARG1 (BioLegend, USA, 165804); CD86 (BioLegend, USA, 105013); M‐CSF (MCE, USA, HY‐P70488); IL4 (MCE, USA, HY‐139092); PMA (MCE, USA, HY‐18739); collagenase Type IV (Sigma, USA, C5138); deoxyribonuclease I (Thermo, USA, EN0521); Matrigel (Corning, USA, 354234); Organoid culture medium (Omabio, China, OM12).

### 2.2 Human Tissue Specimens

Ethical approval for this study was granted by the Ethics Committee of Ruijin Hospital, Shanghai Jiao Tong University School of Medicine (Approval No. 2021 Clinical Ethics 161). Written informed consent was obtained from all individuals enrolled in the study. Due to limited clinical annotation, detailed molecular subtype information was not available for all organoid samples. This limitation has been acknowledged in the Discussion.

### 2.3 Prediction of Drug Targets for Ginsenoside Rb1

For in silico target identification, the SMILES code for ginsenoside Rb1 was obtained from PubChem. Potential targets were systematically screened using three online platforms: SuperPred, Swiss Target Prediction, and PharmMapper.

### 2.4 Prediction of Therapeutic Targets for PDAC

Potential PDAC‐related therapeutic targets were collected from DisGeNET, DrugBank, OMIM, GeneCards, and the Therapeutic Target Database.

### 2.5 Protein–Protein Interaction Network Construction and Functional Enrichment Analysis

A PPI network was generated from the consensus targets using the STRING database with a confidence threshold of > 0.7. Hub genes were determined via network topology analysis, and visualization was performed using Cytoscape 3.9.1. Functional enrichment (GO and KEGG) analyses were performed using the clusterProfiler R package (v4.3.2). This network‐based approach was used to identify potential regulatory nodes and pathways associated with GRB1 activity in PDAC. For GO and KEGG enrichment analyses, *p* values were adjusted using the Benjamini–Hochberg false discovery rate method, and terms with adjusted *p* < 0.05 were considered significant.

### 2.6 Molecular Docking Analysis

The crystal structures of the key targets AKT and PIK3CA were retrieved from the Protein Data Bank (PDB). The PubChem‐derived 2D structure of ginsenoside Rb1 was transformed into a 3D model using ChemDraw software. The binding characteristics between ginsenoside Rb1 and the target proteins were assessed through molecular docking using AutoDock Vina (v1.2.2). All the structures were converted to the PDBQT format following desolvation and polar hydrogen addition. A cubic grid box (30 × 30 × 30 Å) with a spacing of 0.05 nm was centered on each protein's active pocket to maximize ligand sampling. All simulations were performed using AutoDock Vina 1.2.2.

### 2.7 Animal Experiments

SPF male C57BL/6 mice (8 week‐old, 20 ± 2 g; Certificate no. AUP‐2025‐03) were sourced from Shanghai Fennuo Biotechnology Co. Ltd., and maintained at the Experimental Animal Center of Ruijin Hospital, Shanghai Jiao Tong University. To establish an immortalized pancreatic cancer cell line, we obtained KPC mice (Shanghai Model Organisms Center, Cat. No. NM‐KI‐210096) and then derived the cell lines from their tumors. The animals were housed under stable temperature (23°C ± 2°C), humidity (65% ± 5%), and a 12 h light/dark cycle, with free access to food and water.

Mice were randomly divided into five groups (*n* = 10): model (saline, 20 mL/kg/day), positive control (gemcitabine, 20 mg/kg, twice weekly), and GRB1‐treated groups (25, 50, and 100 mg/kg/day). An orthotopic pancreatic cancer model was established by inoculating KPC cells into the mouse pancreas. Body weight and food intake were measured every 3 days. The overall timeline of the study is shown in Figure 1A. All animal procedures were reviewed and approved by the Institutional Animal Care and Use Committee and complied with ARRIVE guidelines. For liver metastasis experiments, KPC‐derived pancreatic cancer cells were used to establish a syngeneic metastatic model in C57BL/6 mice. KPC‐derived cells were co‐injected with untreated or GRB1‐treated M2‐like macrophages to evaluate the effect of macrophage modulation on metastatic burden.

**FIGURE 1:**
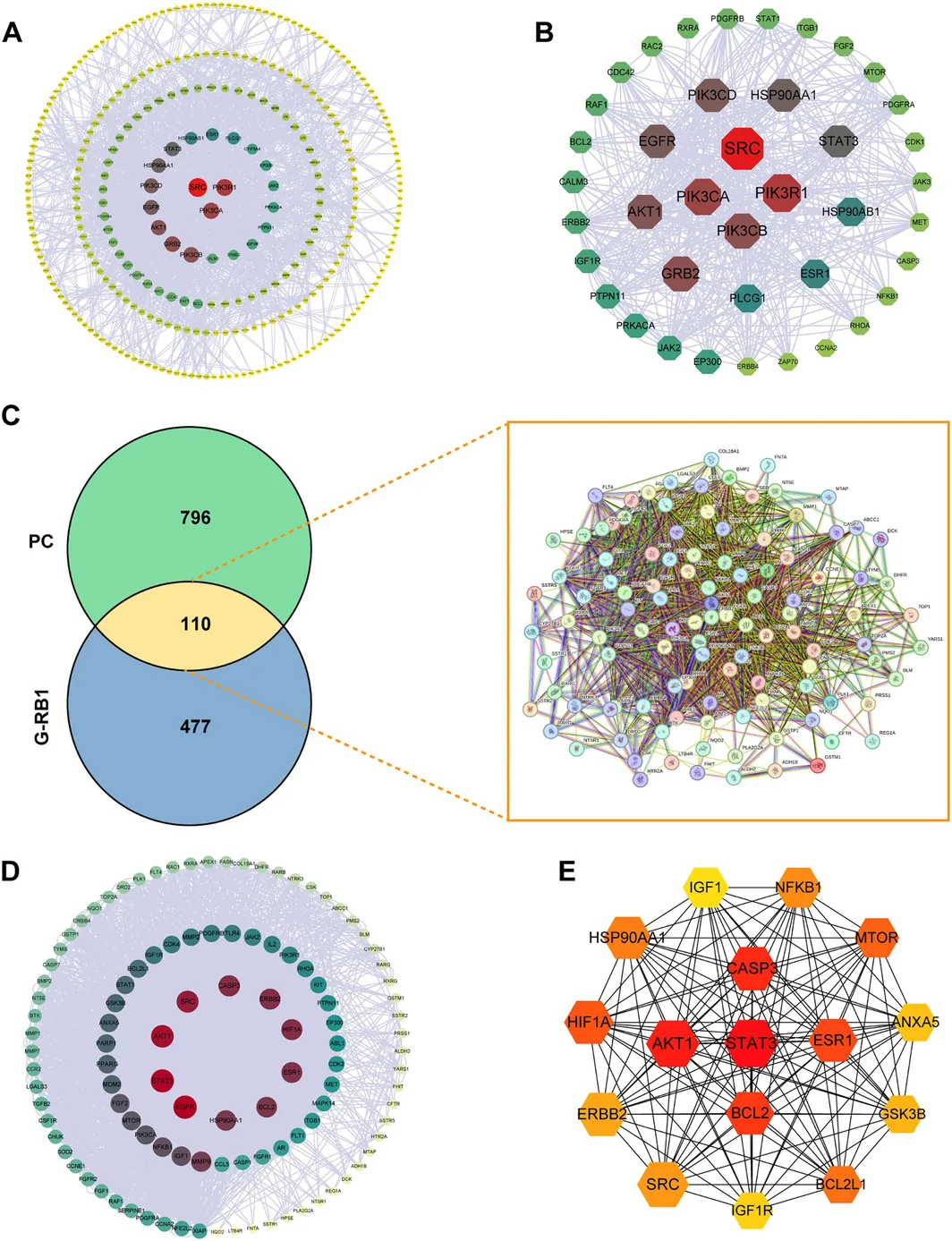
Network pharmacology predicts therapeutic targets of ginsenoside Rb1 (GRB1) in pancreatic cancer. (A) GRB1 target genes predicted using PharmMapper, Swiss Target Prediction, and Super‐PRED databases. (B) Top 20 predicted targets ranked by score. (C) Overlapping targets between GRB1 and pancreatic ductal adenocarcinoma (PDAC). (D) Cytoscape visualization and network topology of shared targets. (E) Top 16 hub genes potentially mediating GRB1's therapeutic effects against PDAC.

Randomization was performed using a random number table. Investigators were blinded to group allocation during tumor measurement and data analysis. Sample size was estimated based on preliminary data using an expected effect size of 0.8, statistical power of 0.8, and *α* = 0.05. No animals were excluded from the analysis. Each in vivo treatment group contained 10 mice, and tumor measurement, histological evaluation, and quantitative image analysis were performed by investigators blinded to group allocation. For immunohistochemical and immunofluorescence analyses, multiple non‐overlapping fields were quantified from each tumor section, and biological replicates were defined as individual animals. Technical replicates were defined as repeated measurements or multiple imaging fields from the same biological sample. Unless otherwise stated, in vitro experiments were independently repeated at least three times.

### 2.8 Histopathological Examination

Histopathological evaluation was carried out on tumor and liver specimens from orthotopic and liver metastasis mouse models. Following collection, the tissues were fixed, paraffin‐embedded, and sectioned. Hematoxylin and eosin staining was used to characterize the overall histopathology, whereas immunohistochemical assays were performed to assess the tumor infiltration indices and macrophage‐specific markers.

### 2.9 IHC

Immunohistochemical analysis of formalin‐fixed paraffin‐embedded (FFPE) tissues was performed following a conventional protocol. The tissue sections were deparaffinized, rehydrated, and subjected to heat‐mediated antigen retrieval. Peroxidase quenching and blocking steps were performed to minimize endogenous activity and nonspecific antibody binding. The sections were subsequently incubated with primary antibodies, followed by detection using an HRP‐conjugated secondary antibody. Chromogenic development was performed with DAB, and the slides were counterstained, dehydrated, and mounted. All runs included appropriate positive and negative controls to confirm staining specificity and reliability.

### 2.10 Quantitative Real‐Time PCR

Total RNA was isolated from cells or tissues using TRIzol reagent and reverse‐transcribed into cDNA using HiScript II Q RT SuperMix following the manufacturer's protocols. qPCR was carried out using SYBR Green Master Mix on an ABI 7500 Fast Real‐Time PCR instrument. qPCR was performed in a 20 μL reaction volume containing 10 μL SYBR Green Master Mix, 0.5 μM of each primer, and 1 μL cDNA template. Cycling conditions were: 95°C for 30 s, followed by 40 cycles of 95°C for 5 s and 60°C for 30 s. Relative mRNA levels were determined using the 2^−ΔΔCt method, normalized to β‐actin.

### 2.11 Western Blotting

Tumor specimens and hepatic metastatic lesions were processed in ice‐cold RIPA buffer supplemented with protease and phosphatase inhibitor cocktails for 30 min. After centrifugation (12,000 × g, 15 min, 4°C), the supernatants were collected and protein concentrations were quantified using a BCA assay. Equal amounts of protein (30 μg) were combined with the loading buffer, denatured by heating, separated by SDS–PAGE, and electrotransferred onto PVDF membranes. Membranes were blocked in 5% nonfat milk or BSA in TBST, then probed with primary antibodies at 4°C overnight, followed by incubation with HRP‐conjugated secondary antibodies for 1 h at room temperature. Protein signals were detected using enhanced chemiluminescence (ECL) and captured using a chemiluminescence detection system.

### 2.12 Immunofluorescence Staining

Immunofluorescent staining was carried out on FFPE tissue sections, which were first baked at 60°C for 1 h prior to deparaffinization and rehydration. Antigen retrieval was performed using a heat‐mediated method in appropriate retrieval buffer. After cooling to room temperature, sections were permeabilized with 0.3% Triton X‐100 for 10 min and blocked with 5% normal goat serum for 1 h. Slides were then incubated with primary antibodies at 4°C overnight, followed by fluorophore‐conjugated secondary antibodies for 1 h at room temperature. Signal quenching between staining cycles allowed sequential antibody application for multiplex detection. Imaging was performed using either a fluorescence microscope or a multispectral imaging platform.

### 2.13 Human Peripheral Blood Mononuclear Cell Isolation

Fresh peripheral blood was collected from healthy donors into anticoagulant tubes after informed consent. PBMCs were isolated by density‐gradient centrifugation using lymphocyte separation medium according to the manufacturer's instructions. The PBMC layer was collected, washed twice with PBS, and resuspended in RPMI 1640 medium supplemented with 10% FBS for subsequent macrophage differentiation experiments.

### 2.14 Flow Cytometry

Fresh tumor specimens were finely minced and digested at 37°C for 30–45 min in an enzyme cocktail containing DNase I (0.1 mg/mL), collagenase IV (1 mg/mL), and 0.25% trypsin with intermittent gentle pipetting to promote tissue dissociation. The digested suspension was passed through a 70‐μm cell strainer to generate a single‐cell preparation and was washed twice with PBS. After counting, 1 × 10^6^ cells were preincubated with Fc‐block (anti‐CD16/32) for 10 min, then labeled with fluorochrome‐conjugated antibodies (e.g., CD45, CD11b, F4/80) for 30 min at 4°C in the dark. Following two PBS washes, the cells were suspended in 2% FBS/PBS and analyzed using a BD FACS Canto II or Cytek Aurora flow cytometer. Data were processed using FlowJo, with tumor‐associated macrophages (TAMs) gated as CD45^+^CD11b^+^F4/80^+^ cells.

### 2.15 Macrophage Polarization

To induce M2‐like polarization, THP‐1 cells were first differentiated with PMA (100 ng/mL) for 48 h and subsequently stimulated with IL‐4 (20 ng/mL) for a further 48 h. PBMC‐derived M2‐like macrophages were generated by allowing cells to adhere for 24 h, differentiating adherent monocytes with M‐CSF (20 μg/mL) for 6 days, and inducing M2 polarization with IL‐4 (20 ng/mL).

### 2.16 Patient‐Derived Organoid Culture and Macrophage‐Conditioned Medium Assay

Patient‐derived pancreatic tumor organoids were established from one surgically resected PDAC specimen after written informed consent. Organoids were embedded in Matrigel and maintained in organoid culture medium according to the manufacturer's instructions. For macrophage‐conditioned medium assays, conditioned medium was collected from untreated M2‐like macrophages or GRB1‐treated M2‐like macrophages and applied to organoid cultures. Organoid growth was assessed by bright‐field imaging, and organoid area was quantified using ImageJ. Each condition included at least three technical replicates, and the experiment was independently repeated three times using the same organoid line. Because only one patient‐derived organoid model was used, inter‐patient heterogeneity could not be assessed.

### 2.17 Statistical Analysis

Statistical analyses were performed using GraphPad Prism v9.0. Normality was assessed using the Shapiro–Wilk test. Normally distributed data are presented as mean ± SEM and were analyzed using one‐way ANOVA followed by Dunnett's multiple‐comparison test. Non‐normally distributed data are presented as median with interquartile range and were analyzed using appropriate non‐parametric tests. A two‐sided *p* < 0.05 was considered statistically significant. Biological and technical replicates are indicated in the corresponding figure legends.

## 3 Results

This study found the food‐derived bioactive compound ginsenoside Rb1 (GRB1) inhibits pancreatic cancer progression by reprogramming tumor‐associated macrophages (Graphical abstract). GRB1 inhibits the PI3K‐AKT signaling pathway, thereby blocking the polarization of M0 to M2 macrophages and reducing the accumulation of immunosuppressive macrophages in the tumor microenvironment. These results provide experimental support for the anti‐PDAC potential of a bioactive constituent derived from a medicine–food homologous botanical.

### 3.1 Network Pharmacology Identifies Potential GRB1‐Associated Targets in Pancreatic Cancer

To identify potential GRB1‐associated targets in pancreatic cancer, we integrated data from Swiss Target Prediction, SuperPred, and PharmMapper and identified 587 candidate GRB1 targets (Figure 1A,B). In parallel, 905 pancreatic cancer‐related targets were collected from GeneCards, DisGeNET, DrugBank, OMIM, and TTD. Intersection analysis identified 110 shared targets, which were visualized using a Venn diagram and a target interaction network (Figure 1C). The PPI network contained 491 nodes and 3728 edges (Figure 1D). Based on degree centrality, betweenness centrality, and closeness centrality, 90 targets exceeded the average values for all three parameters. The top hub targets included STAT3, AKT1, CASP3, ESR1, BCL2, HIF1A, HSP90AB1, IGF1, NFκB1, MTOR, ANXA5, GSK3B, BCL2L1, IGF1R, SRC, and ERBB2 (Figure 1E). Together with the KEGG enrichment analysis described below, this screening pipeline progressively narrowed the candidate space and ultimately locked PI3K‐AKT signaling as the priority pathway mediating the anti‐PDAC activity of GRB1. The predicted targets serve as hypotheses generation; experimental validation was focused on the PI3K‐AKT pathway based on KEGG enrichment ranking.

### 3.2 Food‐Derived Bioactive Compound Ginsenoside Rb1 Suppresses Pancreatic Cancer Progression In Vivo

We next evaluated the therapeutic effect of GRB1 in an orthotopic PDAC mouse model established using KPC‐derived cells (Figure 2A,B). Compared with the model group, GRB1 treatment reduced tumor growth, with the high‐dose group showing the most pronounced effect and an efficacy comparable to gemcitabine (Figure 2C–E). Ki67 staining further supported reduced tumor cell proliferation after GRB1 treatment (Figure 2F,G). High‐dose GRB1 also prolonged survival without obvious body weight loss (Figure 2H).

**FIGURE 2:**
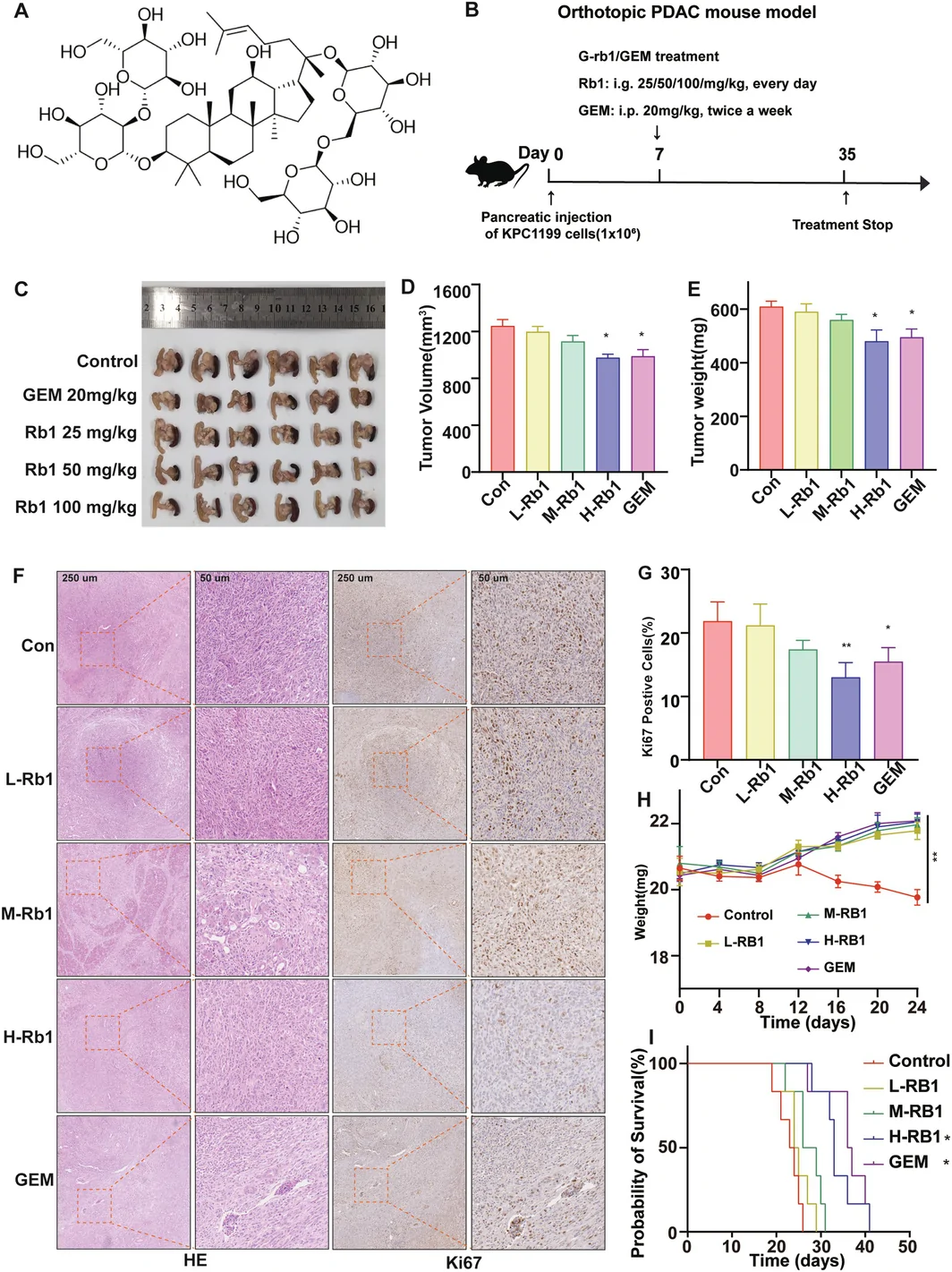
Food‐derived bioactive compound GRB1 suppresses pancreatic tumor growth in vivo. (A) Chemical structure of GRB1. (B) Experimental design of the orthotopic pancreatic cancer mouse model and GRB1 treatment. (C) Representative tumor images from control and GRB1‐treated mice. (D, E) Tumor volume and final weight following different GRB1 doses. (F, G) H&E and Ki67 staining with quantification showing reduced proliferation after GRB1 treatment. (H) Body weight monitoring indicating no systemic toxicity. (I) Kaplan–Meier curves showing prolonged survival in high‐dose GRB1–treated mice. Data are presented as mean ± SEM. ns, no significance; *p < 0.01, **p < 0.001, ***p < 0.0001.

### 3.3 Food‐Derived Bioactive Compound Ginsenoside Rb1 Reduces Pancreatic Cancer Liver Metastasis

To determine whether GRB1 suppresses pancreatic cancer liver metastasis, we established a liver metastasis model using KPC‐derived pancreatic cancer cells (Figure 3A). GRB1 treatment reduced liver metastatic burden, as shown by smaller tumor‐invading areas, fewer metastatic lesions on H&E staining, and a lower liver‐to‐body weight ratio (Figure 3B–E). GRB1 also reduced the expression of metastasis‐related markers, including Zeb‐1 and Vimentin, whereas E‐cadherin was not significantly increased (Figure 3F,G).

**FIGURE 3:**
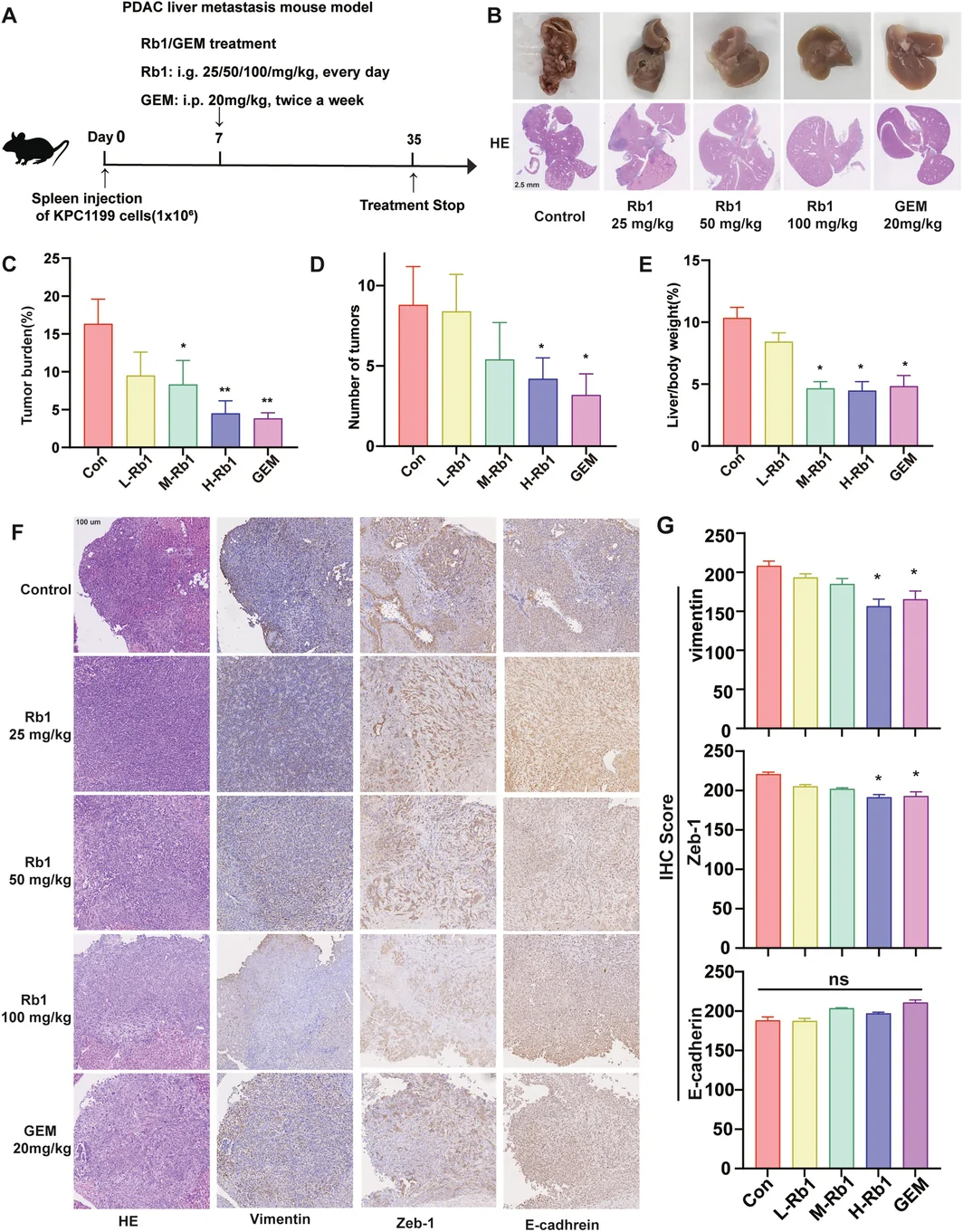
Food‐derived bioactive compound GRB1 inhibits hepatic metastasis of pancreatic ductal adenocarcinoma (PDAC). (A) Experimental scheme of the PDAC liver metastasis model and GRB1 or gemcitabine (GEM) treatment. (B) Representative liver images and H&E staining showing reduced metastatic nodules after GRB1 or GEM treatment. (C–E) Quantification of tumor burden, metastatic nodule number, and liver‐to‐body weight ratio. (F, G) H&E and IHC staining for EMT markers (Vimentin, Zeb‐1, E‐cadherin) and semi‐quantitative analysis showing that GRB1 reduced Vimentin and Zeb‐1 expression, indicating EMT suppression in liver metastases. Data are presented as mean ± SEM. ns, no significance; *p < 0.01, **p < 0.001, ***p < 0.0001.

### 3.4 Food‐Derived Bioactive Compound GRB1 Treatment Is Associated With Reduced PI3K‐AKT Signaling

We next examined potential molecular pathways associated with GRB1 activity. GO and KEGG enrichment analyses were performed using the intersecting targets of GRB1 and PDAC (Figure 4A). GO terms were enriched in kinase activity regulation, MAPK cascade initiation, gland morphogenesis, phosphatidylinositol‐related signaling, receptor tyrosine kinase activity, and protein/phosphatase binding. KEGG analysis identified PI3K‐AKT signaling as one of the most enriched pathways, suggesting that this pathway may be involved in GRB1‐associated anti‐PDAC activity (Figure 4B).

**FIGURE 4:**
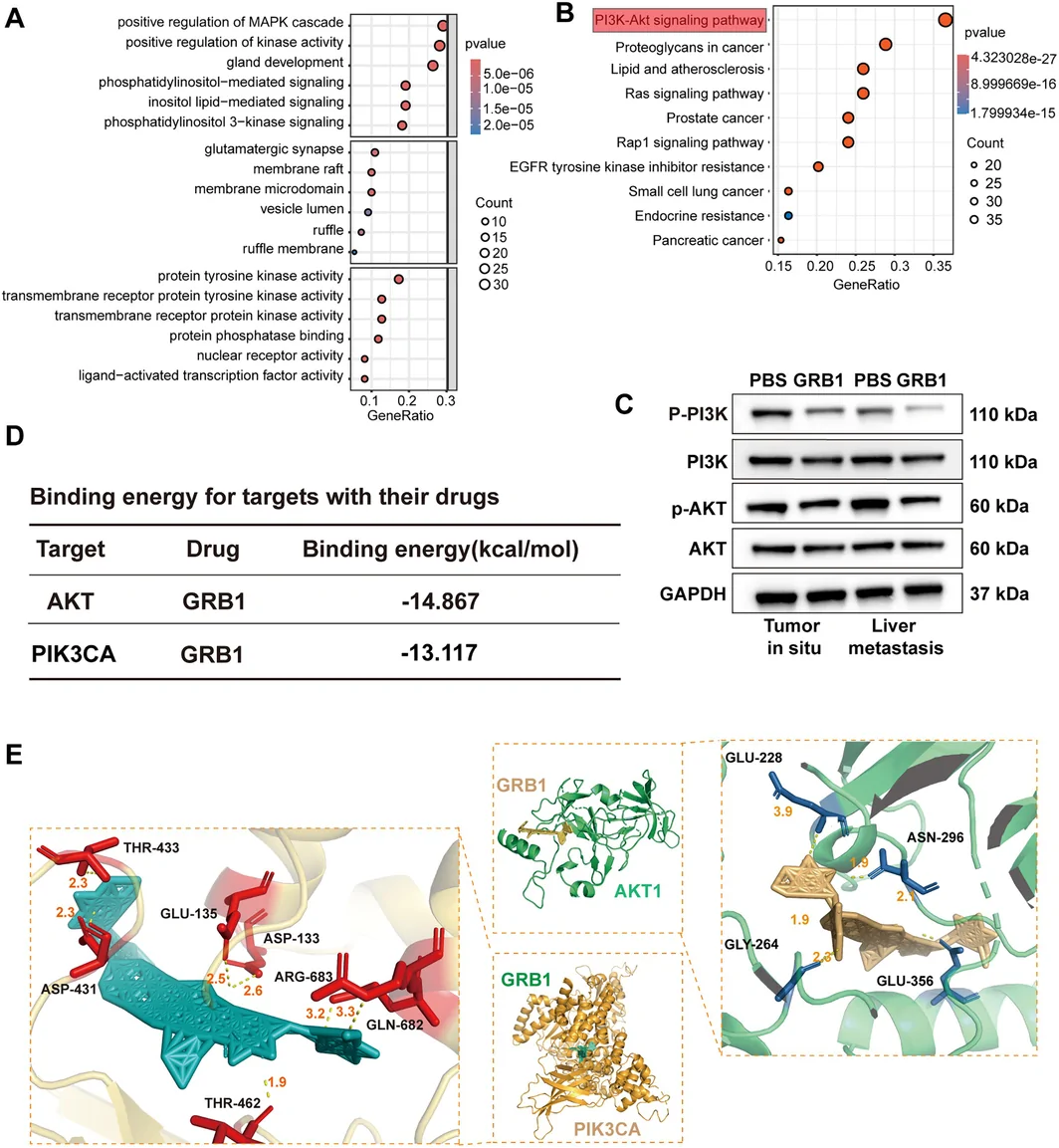
GRB1 suppresses pancreatic cancer progression by targeting the PI3K–AKT signaling pathway. (A) GO enrichment analysis of GRB1–PDAC overlapping targets showing enrichment in kinase regulation and phosphatidylinositol signaling. (B) KEGG enrichment highlighting the PI3K–AKT pathway as the key signaling axis. (C) Western blot showing decreased phosphorylation of PI3K and AKT in GRB1‐treated tumor and liver metastasis tissues. (D, E) Molecular docking and visualization demonstrating stable binding of GRB1 to AKT1 and PIK3CA with strong binding energies. Data are presented as mean ± SEM. ns, no significance; *p < 0.01, **p < 0.001, ***p < 0.0001.

To examine this possibility, we assessed PI3K‐AKT pathway components by western blotting. GRB1 reduced PI3K and AKT phosphorylation in PDAC cells in a dose‐dependent manner (Figure 4C). Molecular docking further suggested possible interactions between GRB1 and pathway‐related proteins, including AKT1 and PIK3CA (Figure 4D,E). Together, these data suggest that GRB1 treatment is associated with reduced PI3K‐AKT signaling in PDAC models, although this pathway cannot be considered the exclusive mediator of GRB1 activity based on the current evidence.

### 3.5 Food‐Derived Bioactive Compound Ginsenoside Rb1 Reduces M2‐Like Tumor‐Associated Macrophage Features in Pancreatic Cancer Mice

Although GRB1 showed antitumor activity in vivo, it exerted limited direct cytotoxicity in pancreatic cancer cell lines, and no obvious cytotoxicity was observed in the tested normal pancreatic cell line under the experimental conditions (Figure 5A and Figure S1). These findings suggest that GRB1 may act partly through modulation of the TME. Because macrophages are abundant in the PDAC microenvironment, we examined whether GRB1 affected macrophage‐associated immune features.

**FIGURE 5:**
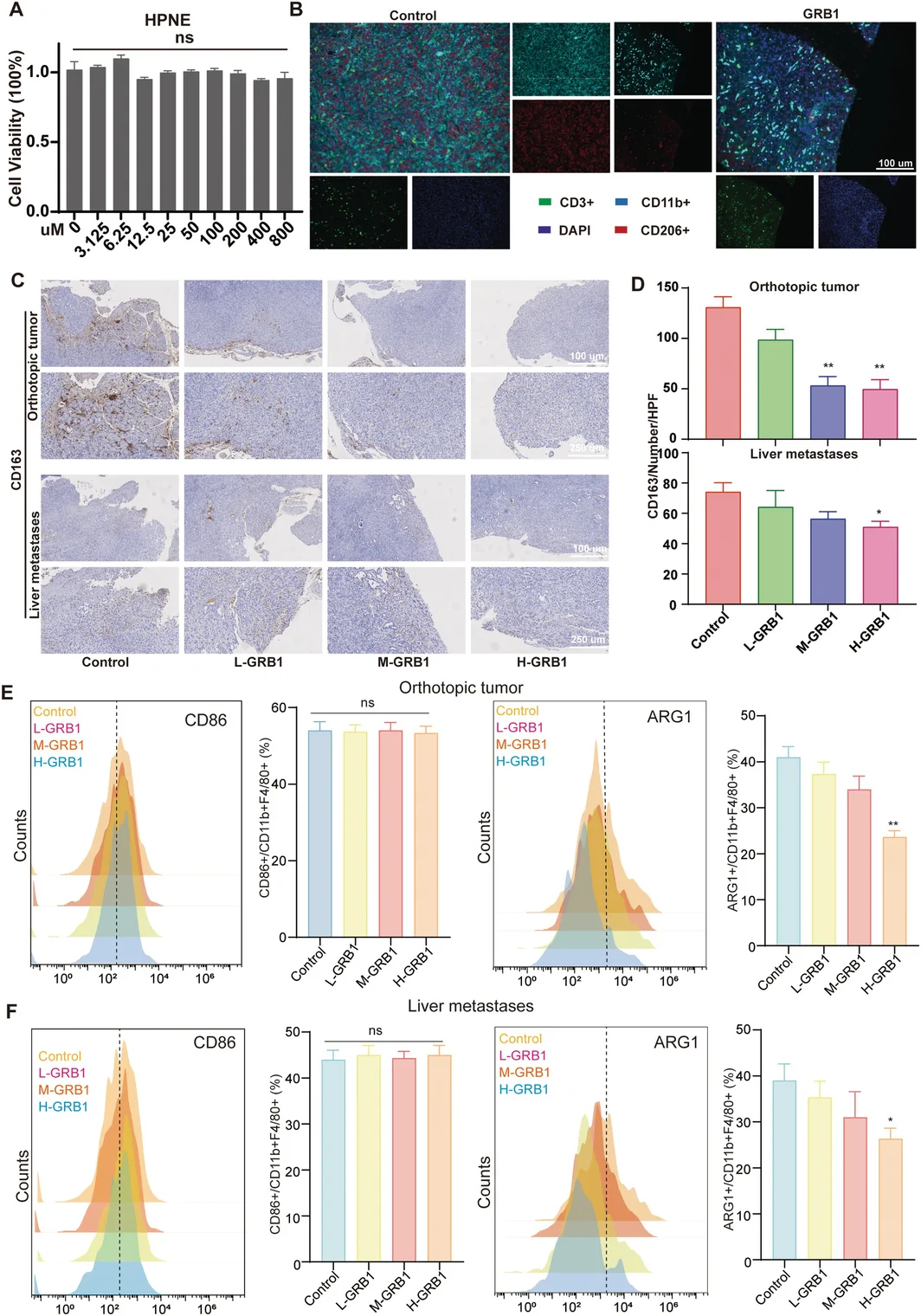
GRB1 suppresses M2 macrophage polarization in the pancreatic tumor microenvironment. (A) GRB1 showed no cytotoxicity toward normal pancreatic ductal epithelial HPNE cells. (B) Multiplex immunofluorescence staining revealed reduced CD206^+^ macrophage infiltration in GRB1‐treated tumors. (C, D) IHC staining and quantification of CD163 in orthotopic and liver metastatic tumors showing dose‐dependent inhibition of M2 macrophage accumulation. (E, F) Flow cytometry of macrophages from orthotopic and metastatic tumors showing that GRB1 decreased ARG1^+^CD11b^+^F4/80^+^ M2 macrophages but not CD86^+^ M1 macrophages, indicating selective suppression of M2 polarization. Data are presented as mean ± SEM. ns, no significance; *p < 0.01, **p < 0.001, ***p < 0.0001.

Multiplex immunofluorescence showed that macrophages represented a major immune cell population in pancreatic tumor tissues, and the most notable changes after GRB1 treatment were observed in M2‐like macrophage markers (Figure 5B and Figure S2). IHC and flow cytometry further showed that GRB1 reduced M2‐like macrophage polarization in a dose‐dependent manner in both orthotopic and liver metastasis models (Figure 5C–F). The markers used here, including CD206, CD163, ARG1, CD115, and TGFB1, are commonly used indicators of M2‐like polarization but do not fully capture TAM heterogeneity in PDAC.

### 3.6 Food‐Derived Bioactive compound GRB1‐Treated M2‐Like Macrophages are Associated With Reduced Tumor‐Promoting Activity in PDAC Models

We next examined whether GRB1 directly modulates macrophage polarization. THP‐1‐derived macrophages and PBMC‐derived monocyte‐derived macrophages were used to generate M2‐like macrophages (Figure 6A). GRB1 had minimal effects on unpolarized macrophages but reduced the induction of M2‐like polarization. GRB1 also decreased the expression of M2‐like markers, including CD206, CD115, and TGFB1, in both macrophage models (Figure 6B). Western blotting showed reduced PI3K and AKT phosphorylation in GRB1‐treated M2‐like macrophages, suggesting that reduced PI3K‐AKT signaling may be associated with GRB1‐mediated macrophage modulation (Figure 6C).

**FIGURE 6:**
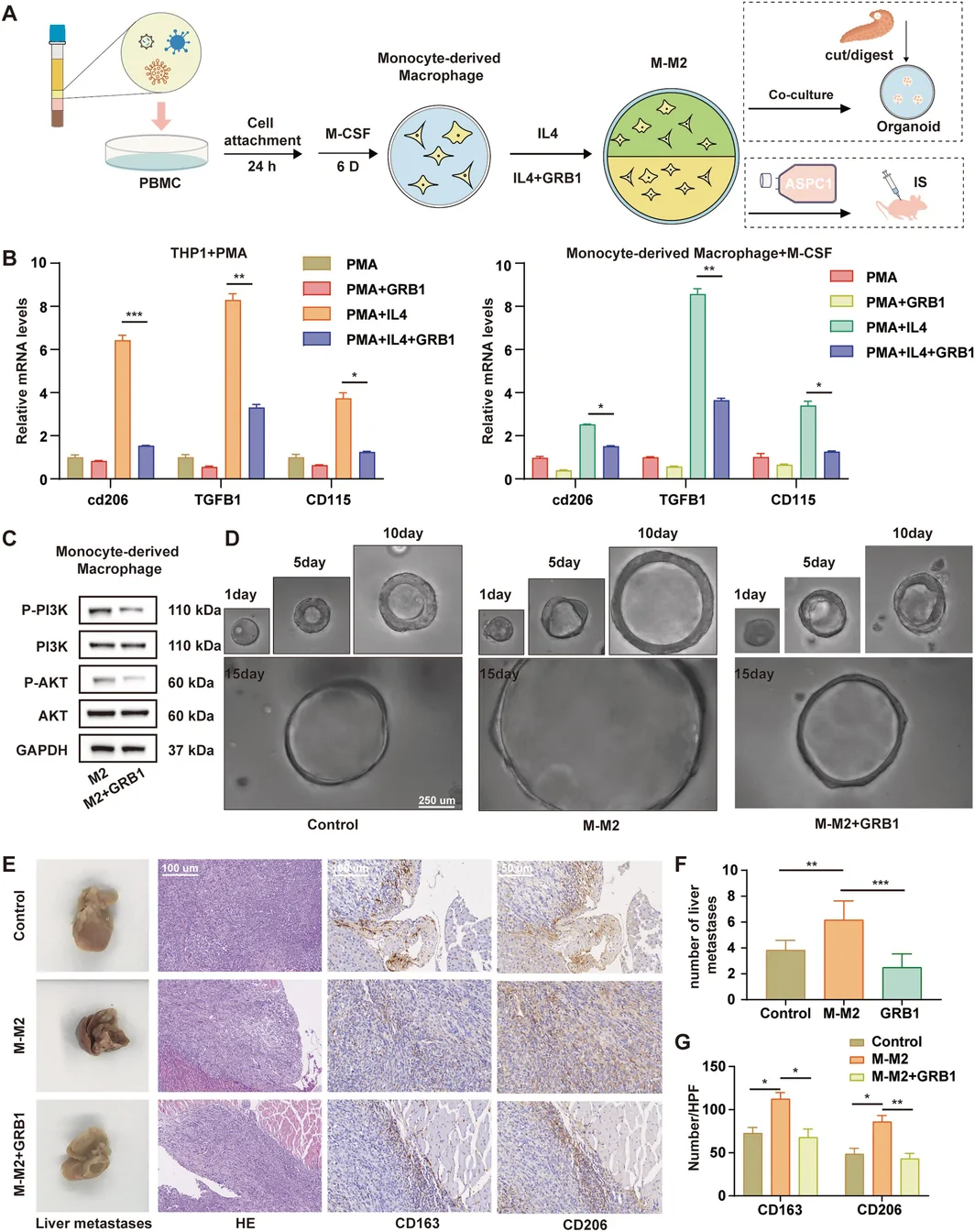
Food‐derived bioactive compound GRB1 inhibits M2 macrophage polarization and pancreatic cancer progression via PI3K–AKT signaling. (A) Schematic of macrophage differentiation and co‐culture workflow. PBMCs or THP‐1 cells were induced to M2 macrophages with IL‐4, with or without GRB1 treatment. GRB1‐treated macrophages were co‐cultured with pancreatic cancer organoids or co‐injected with ASPC1 cells in vivo. (B) qRT–PCR showing reduced expression of M2 markers (CD206, TGFB1, CD115) after GRB1 treatment. (C) Western blot showing that GRB1 inhibited PI3K and AKT phosphorylation in macrophages. (D) Bright‐field images of organoids co‐cultured with control, M2, or GRB1‐treated M2 macrophages, showing reduced growth in the GRB1 group. (E–G) Representative liver images, H&E/IHC staining for CD163 and CD206, and quantification showing that GRB1 decreased M2 macrophage infiltration and liver metastases. Data are presented as mean ± SEM. ns, no significance; *p < 0.01, **p < 0.001, ***p < 0.0001.

In one patient‐derived pancreatic tumor organoid model, conditioned medium from GRB1‐treated M2‐like macrophages reduced organoid growth compared with conditioned medium from untreated M2‐like macrophages (Figure 6D). As a macrophage functional validation model, KPC‐derived pancreatic cancer cells were co‐injected with untreated or GRB1‐treated M2‐like macrophages in C57BL/6 mice. Co‐injection with GRB1‐treated M2‐like macrophages was associated with reduced metastatic burden (Figure 6E,F). These findings suggest that GRB1‐treated macrophages have reduced tumor‐promoting activity. However, whether PI3K‐AKT inhibition is required for this effect remains to be determined.

### 3.7 In Vivo Effects of Food‐Derived Bioactive compound GRB1 on Organ Toxicity and Systemic Inflammation

We next evaluated the in vivo safety and systemic tolerability of GRB1. To assess potential organ toxicity, we examined major organs (lung, heart, kidney, liver, and spleen) and quantified the liver‐to‐body weight ratio in control and GRB1‐treated mice (Figure 7A). No gross morphological abnormalities or significant differences in liver/body weight were observed between groups. Serum biochemical parameters reflecting hepatic and renal function, including alanine aminotransferase (ALT), aspartate aminotransferase (AST), blood urea nitrogen (BUN), creatinine (CREA), and uric acid (UA), remained unchanged after GRB1 treatment (Figure 7B). Furthermore, hepatic mRNA expression levels of pro‐inflammatory cytokines (Tnf‐α, Ifn‐γ, and Il6) were not significantly altered by GRB1 administration (Figure 7C). Collectively, these results indicate that GRB1 does not elicit overt organ damage or systemic inflammation under the tested conditions.

**FIGURE 7:**
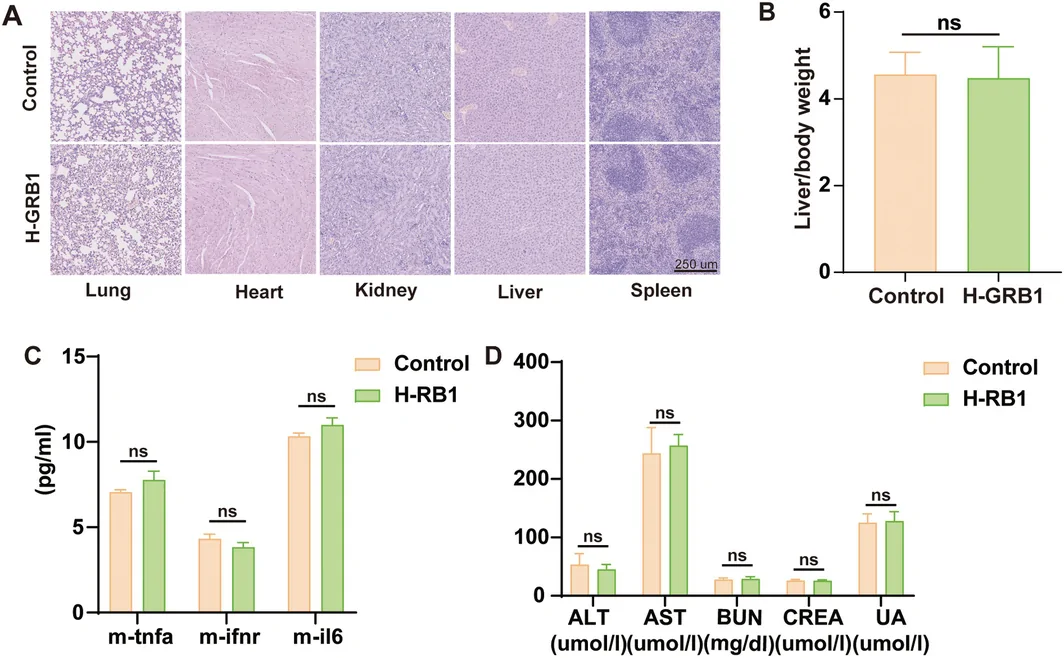
In vivo safety evaluation of food‐derived bioactive compound Ginsenoside Rb1. (A) Representative H&E staining of major organs (lung, heart, kidney, liver, spleen) from control and high‐dose GRB1 (H‐GRB1)‐treated mice. Scale bar, 250 μm. (B) Liver‐to‐body weight ratio in control and H‐GRB1 groups. (C, D) Serum biochemical parameters reflecting liver and kidney function: alanine aminotransferase (ALT), aspartate aminotransferase (AST), blood urea nitrogen (BUN), creatinine (CREA), and uric acid (UA). Data are presented as mean ± SEM; ns, no significant.

## 4 Discussion

Chai‐Gui‐Xiao‐Ji Formula is a commonly used prescription with significant clinical efficacy. Other components in the formula also contribute to its efficacy, among which GRB1 is the most representative food‐derived monomer. This study provides preclinical evidence thae food‐derived bioactive compound GRB1 suppresses PDAC progression and modulates the immunosuppressive tumor microenvironment. Using a network pharmacology‐guided strategy, we identified PI3K‐AKT signaling as a potential GRB1‐associated pathway. Experimental validation showed that GRB1 reduced PI3K and AKT phosphorylation, inhibited primary tumor growth, and reduced liver metastasis. These findings suggest that GRB1 may exert anti‐PDAC activity partly through PI3K‐AKT‐associated signaling. Viewed through the lens of medicine–food homology, these findings illustrate how a dietary botanical constituent can engage tumor‐immune signaling in a manner compatible with long‐term nutritional intervention. While the current study focused on PI3K‐AKT, the enrichment analysis also identified other pathways; their involvement cannot be excluded and warrants further investigation.

A major finding of this study is the food‐derived bioactive compound GRB1 treatment was associated with reduced M2‐like features of tumor‐associated macrophages. TAMs are important components of the PDAC TME and contribute to tumor growth, metastasis, and therapeutic resistance (Y. Chen, Song, et al. 2019; Hindson 2024). GRB1 reduced M2‐like macrophage markers in tumor tissues, THP‐1‐derived macrophages, and PBMC‐derived macrophages. Functional assays using macrophage‐conditioned medium and one patient‐derived organoid model further suggested that GRB1‐modulated macrophages may have reduced tumor‐promoting activity. However, the causal link between PI3K‐AKT inhibition and macrophage modulation remains to be confirmed by pathway rescue or genetic gain‐of‐function studies.

Immunotherapy has shown significant efficacy in various solid tumors, but its clinical application in pancreatic ductal adenocarcinoma (PDAC) has yielded little success so far (Lou et al. 2026). Therefore, finding effective ingredients from natural foods is a better strategy. GRB1 is a principal bioactive constituent of *Panax ginseng* , a medicinal herb with a long history of traditional use (G. Zhou et al. 2023), and importantly, an officially recognized medicine–food homology substance that is consumed daily as a food ingredient (Chen et al. 2019b; Ichim and de Boer 2020; Park 2024). Other ginsenosides, such as Rg1 (Hsieh et al. 2023), Re (Peng et al. 2012), and Rg3 (Yao and Zhu 2025), have also been reported to exhibit immunoregulatory and anticancer activities, suggesting the possibility of multi‐component effects in ginseng‐derived therapies. The present findings support further investigation of GRB1 as a potential TME‐modulating candidate in PDAC. As a constituent of medicine–food homologous botanicals with a long history of safe consumption, GRB1 offers a safety advantage compatible with chronic dietary intake.

From the perspective of food science and nutrition, these findings carry practical implications. First, the favorable safety profile observed in vivo (Figure S1) is consistent with the long dietary history of ginseng and supports the feasibility of developing GRB1‐enriched ginseng products as functional foods or medical foods for individuals at high risk of PDAC or as adjuvants during therapy. Second, the clinical translation of GRB1 as a food‐derived agent requires systematic investigation of its oral bioavailability, gut microbiota‐mediated deglycosylation to compound K, food‐matrix effects, and processing stability, all of which are central topics in food and nutrition research. Third, given that PDAC is tightly linked to dietary and metabolic factors (Chan et al. 2022; Zhang et al. 2025), integrating ginseng‐derived functional ingredients into evidence‐based nutritional intervention strategies may open a preventive, low‐toxicity avenue complementary to conventional oncologic treatment. Together, these considerations underscore the translational value of the medicine–food homology framework for developing functional food products in oncology nutrition.

In 2025, a research team led by Chang‐Sheng Dong from Shanghai University of Traditional Chinese Medicine found that ginsenoside Rb1 achieves anti‐pancreatic cancer effects through a dual pathway of activating myeloid cell immune stimulation and inhibiting cancer‐related neural remodeling, thereby “Tiao‐Yuan‐Jie‐Du” theory. This theory can guide individualized dietary maintenance in food nutrition (Fang et al. 2024; Ruixin et al. 2024; Sun et al. 2025), selecting foods based on constitutional differences, embodying the concept of “medicine and food are of the same origin”, and adjusting pathological states through daily diet, ultimately achieving the goal of health preservation, disease prevention, and longevity enhancement.

Several limitations should be acknowledged. First, the present study does not establish definitive causality between PI3K‐AKT inhibition and macrophage modulation. Second, TAM characterization was mainly based on conventional M2‐associated markers and cannot fully resolve TAM heterogeneity. Third, only one patient‐derived organoid line was used, and inter‐patient heterogeneity could not be assessed. Fourth, the network‐level analysis was based mainly on target prediction, PPI construction, pathway enrichment, molecular docking, and experimental validation, which do not replace transcriptomic, proteomic, single‐cell, or spatial validation. Finally, the clinical translation of GRB1 requires further pharmacokinetic, bioavailability, biodistribution, formulation, dose‐optimization, and long‐term safety studies. Moreover, the standardization and quality control required for medicine–food homologous products remain to be addressed before functional food development.

The present study used intraperitoneal administration; oral bioavailability and gut microbiota‐mediated metabolism of GRB1 require separate investigation before functional food applications. In the future, necessary steps such as stability research, toxicological evaluation, and GRAS (Generally Recognized as Safe) determination will also be required.

## 5 Conclusion

In conclusion, starting from the medicine–food homology herb *Panax ginseng* , a combined UPLC‐Q‐TOF‐MS and network pharmacology screening strategy locked GRB1 as the core anti‐PDAC candidate; GRB1 showed potential anti‐PDAC activity and was associated with reduced PI3K‐AKT signaling and decreased M2‐like macrophage polarization. These findings suggest that GRB1 may modulate the immunosuppressive PDAC microenvironment and support its development as a ginseng‐derived functional food ingredient and nutritional adjuvant for PDAC prevention and supportive care, pending further bioavailability and clinical studies. This work thus provides a scientific paradigm for translating medicine–food homologous botanicals into evidence‐based functional foods for cancer nutritional support.

## Author Contributions

**Chang‐Sheng Dong:** conceptualization, supervision, funding acquisition, writing – original draft, formal analysis, data curation. **Gaoqiang Wang:** conceptualization, data curation, formal analysis, writing – original draft. **Ying Niu:** conceptualization, writing – original draft, formal analysis, data curation. **Xiuqiao Sun:** data curation, formal analysis, writing – original draft. **Wenyi Ren:** writing – original draft. **Jiliang Zhang:** investigation, methodology, visualization. **Yi Zhang:** supervision, project administration. **Rui‐Xin Wu:** supervision, funding acquisition, writing – review and editing. **Xiaoxiong Wu:** software, validation, funding acquisition, resources, writing – review and editing.

## Funding

This work was supported by the Key Discipline Fund of Yangpu Hospital, School of Medicine, Tongji University, yzxxk202608. the Science and Technology Development Project of Shanghai University of Traditional Chinese Medicine, 24YJS08. Wu Jieping Medical Foundation Research Project, 320.6750.2025‐13‐120. National Administration of Traditional Chinese Medicine, ZYYZDXK‐2023063. National Program for the Construction of Advantageous Traditional Chinese Medicine Specialty, XH40202‐20250904. Third Traditional Chinese Medicine Inheritance Talent Project of Shanghai Municipal Hospital of Traditional Chinese Medicine, SZYCC(2023‐2024)‐ZYCCXM03. Shanghai Qihuang Ankang Science and Technology Innovation Action Plan Special Project, QHAK20240618. Shanghai Traditional Chinese Medicine Anti‐aging and Holistic Health Science and Technology Innovation Program, 20240618001.

## Conflicts of Interest

The authors declare no conflicts of interest.

## Supporting information


**Figure S1:** In vitro cytotoxicity of food‐derived bioactive compound GRB1 on cell lines.
**Figure S2:** Purity validation of macrophage populations by flow cytometry.

## Data Availability

The data that support the findings of this study are available from the corresponding author upon reasonable request.
