# Supplementary material for: Ginsenoside Rb1 From the Functional Food Panax ginseng Suppresses Pancreatic Cancer Progression via Macrophage Reprogramming Through the PI3K‐AKT Pathway
Source: Food Sci Nutr. 2026 Oct 8;14(10):e72468. doi: 10.1002/fsn3.72468 (PMC13650271; doi:10.1002/fsn3.72468)
Supplement: Supplementary file 1 [file FSN3-14-e72468-s001.docx]

FIGURE S1. In vitro cytotoxicity of food-derived bioactive compound GRB1 on cell lines

Relative cell viability of PANC1, 8988, Mia‑paca2, CFPAC‑1, and ASPCA1 cells treated with increasing concentrations of GRB1 (1–800 μg/mL) for 72 h. Viability was normalized to untreated control (set as 1.0). Across all tested cell lines and concentrations, the relative values remained close to 1.0 (ranging from 1.00 to 1.09), indicating that GRB1 does not exert significant direct cytotoxicity on these pancreatic cancer cells. Data are presented as mean ± SEM; ns, not significant compared with control (one‑way ANOVA with Dunnett’s post‑test).

FIGURE S2. Purity validation of macrophage populations by flow cytometry

Flow cytometric analysis of CD45 and F4/80 expression on the monocyte‑derived macrophages used for polarization assays (as in Fig. 6A). The table shows the percentage of CD45⁺F4/80⁺ double‑positive cells for each individual sample (n = 64). All examined samples exhibited 100% positivity for both markers, indicating a highly pure macrophage population (>99%) with negligible contamination by other cell types. SSC‑A, side scatter‑area. These data confirm the homogeneity and reliability of the macrophage preparations employed in the subsequent functional experiments.
